# Teachers With Special Needs. De-Psychiatrization of Children in Schools

**DOI:** 10.3389/fsoc.2021.781057

**Published:** 2021-12-08

**Authors:** Laura Batstra, A.C. Marieke van Roy, Ernst D. Thoutenhoofd

**Affiliations:** ^1^ Department of Child and Family Welfare, The University of Groningen, Groningen, Netherlands; ^2^ Department of Education and Special Education, The University of Gothenburg, Gothenburg, Sweden

**Keywords:** education, psychiatrisation, special educational needs, inclusive education /schools, teacher competence, teacher agency

## Abstract

Psychiatrization not only affects adults. Ever more children in Western countries are being diagnosed with a mental disorder of behavior, such as ADHD. Children may often be labelled with the best intentions, for example in order to be able to provide them with suitable care and guidance. However, this labelling can have exclusionary effects and often entails the consequence that important discussion about contextual factors that give rise to (the perception of) unwelcome behavior or academic underperformance rarely, if at all, takes place. In this article we contend that although children are of central concern to schools and the design of pupils’ education, it is important not to make pupils the sole owner of problems that arise. It is therefore high time that a far more critical normative stance towards inclusive education is taken, in which the presently widespread biomedical approach is met with a school community response that focuses not on the nature of individual disorders but on the special need for additional capacity that *schools* and *teachers* have in meeting (perceived) deviant behaviors and emotions and/or academic underperformance. We argue that teaching should not set out to remedy individual diagnoses, but that teachers should be supported to extend their professional competence to the benefit of all pupils.

## Introduction

Psychiatrization not only affects adults. Compared with adult mental health care, the mental health care of young people in Western countries has increased even more rapidly (Olfson et al., 2014; Steinhausen, 2015). Epidemiological studies estimate that one in eight children nowadays meet criteria for a mental disorder (Polanczyk et al., 2015; Barican et al., 2021). Parent surveys in the US found child diagnosis rates of 9.5% for ADHD, 7.4% for behavioral/conduct disorders, 7.1% for anxiety, 3.2% for depression, and 2.5% for autism spectrum disorder (Ghandour et al., 2019; Zablotsky et al., 2019). The vast majority of these diagnosed children exhibit mild to moderate problems, while only around 10% of cases are perceived as severe. Despite that, the sharp rise in childhood psychiatric diagnoses has coincided with increased psychotropic medication use among children. A recent meta-analysis on the annual pediatric psychotropic drug prescription prevalence reports global estimates of 15.3% for ADHD medications, 6.4% for antidepressants and 5.5% for antipsychotics (Piovani et al., 2019).

Concerns about the long term safety of medication, overtreatment and overdiagnosis of youths have increased alongside the rapid rise in child psychiatric classifications and treatments (Frances and Batstra, 2013; Rapoport, 2013; Barnett et al., 2020). Especially in the large group of children and youngsters with mild to moderate problems, the benefits of a classification—such as greater understanding and support—may not outweigh its potential harms, like stigma, self-stigma, underperformance due to self-fulfilling prophecies, and side-effects of medical treatment (Batstra et al., 2012). While diagnosis can promote social identification and acceptance, and children themselves sometimes actively engage in their own psychiatrization, it can also lead to social alienation, invalidation and stigmatization (O’Connor et al., 2018; Beeker et al., 2020). Psychiatric diagnoses in youth are associated with social exclusion in later life (Ringbom et al., 2021). A final drawback of diagnostic inflation is that expansive diagnostic procedures and specialized treatments for mild problems entail problematically high youth care costs and draw resources away from severely troubled children and families, who need it the most.

Various scholars (e.g., Timimi, 2015) and governments (e.g., Health Council of the Netherlands, 2014) appeal for the demedicalization and normalization of child emotional and behavioral problems. If we do wish to turn the tide on the rising rate of childhood mental disorder diagnoses, one of the first places to start are schools. Schools and teachers often play an important role in initiating the first steps toward psychiatric assessments and treatments of children (Sax and Kautz, 2003; Harwood and Allen, 2014; Russell et al., 2016; Wienen, 2019). This article takes a closer look at this process and offers one suggestion likely to contribute to less labelling and greater inclusion of children with diverse emotions and behaviors.

## Reification in Schools

Why do teachers tend to suspect that a psychiatric disorder is present in a child that underperforms and/or exhibits challenging or internalizing behavior? There are countless variables present in children and their environments, in teachers, teaching and school environments, and in educational systems generally, that are at least as influential as children’s mental states. One answer to the question is the widespread tendency to mistake a confirmed diagnosis for an explanation for the problems at hand: this factor plays an important role in the rise in childhood psychiatric diagnosis. The process behind this is called reification, which literally means making a thing out of something that lacks object qualities. In the case of psychiatric disorders, it means that our descriptions and naming of groups of problematic behaviors and emotions—notably the mental disorders listed in the Diagnostic and Statistical Manual of Mental Disorders (DSM), a handbook consulted by psychiatrists and psychologists all over the world—are transformed into concrete neurobiological entities that are believed to cause adverse behaviors and emotions, whereas the latter are in fact merely *described* (Hyman, 2010).

In the words of one of the most influential English philosophers of the 19th century, John Stuart Mill (1806-73), the tendency to reify is the tendency “to believe that whatever received a name must be an entity or being, having an independent existence of its own”. This tendency is strongly present in the now dominant biomedical paradigm (Scull, 2021). A main focus in biomedical research and in biomedical education about disorders is the supposed biological underpinnings of mental disorders. However, despite decennia of expensive brain research with ever better equipment and technologies, not a single biomarker has been found for any of the disorders defined in the DSM (Scull, 2021). Nevertheless, publication bias in favour of positive study results (Glasziou and Chalmers, 2017) push ambitious brain researchers to exaggerate their findings. Small detected average group differences are reported as if they apply to every single person with a disorder (Meerman et al., 2020). This so-called ecological fallacy, or the erroneous generalization of a mean group difference to the individual, is both widespread and persistent.

In today’s demanding school environments, hitting upon a suitable neurobiological label that is thought to explain underperformance and deviant behaviors can be a godsend. When a psychiatric diagnosis is made in a child, underachievement and challenging behaviors can be attributed to the disorder, removing guilt and responsibility from teachers, parents and pupils (Wienen et al., 2019). This makes room for a new starting point in the dialogue between parents, teachers and children, along with a shared disorder language and new intervention ideas (Honkasilta et al., 2016). While this collaboration may in principle benefit children, it also makes them the sole owner of problems that in fact arose in a specific context. In addition, an individual diagnosis may create the spurious impression that the cause of problems has been identified. This in turn may stop teachers from trying to find the underlying issues or triggers for problematic behavior or poor academic performance, so that the impact of contextual factors on those remains hidden and in place.

## Inclusive Education: Two Models

The dominance of individual over contextual approaches is also visible in the application of inclusive education (Wienen, 2019). Inclusive education is the policy ideal that all children receive education at a regular school. This ideal is defined in the UNESCO Salamanca Statement and Framework for Action (UNESCO, 1994, p. 8). Regular schools with an inclusive orientation are seen as the most effective means of combating discriminatory attitudes, creating welcoming communities, building an inclusive society, and achieving education for all.

Roughly speaking, two different approaches to inclusive education can be identified: the biomedical and the community approach (Wienen, 2019). The first takes individual children with a disorder or disability as the starting point and is based on the notion that either education must be adapted to enable each child to attend school, or that individual children need specialist help in adjusting to a life in school. The approach essentially presupposes that a gap exists between how some children are and what school expects of them, and that specialists are needed to try and close that gap. The second approach on the other hand, takes education in context and community life as starting points. The focus is on organizing educational context in such a way that every unique child can flourish and be educated, so that special educational needs cease to exist. This alternative approach essentially presupposes and values diversity, so that good fit between children and schooling is inevitably a matter of constant mutual adjustment, involving the entire school community in making suitable accommodations and enable diverse childhoods. Moreover, the approach facilitates the idea that the ability to adjust to and accommodate difference is a skill that is commensurate with—and perhaps even essential to—the exercise of democracy and the practice of tolerance. Worth noting perhaps is that the distinction between biomedical and community approaches seems to reduplicate a distinction between categorical and relational perspectives that has long been commonplace in Nordic reasoning about inclusive education ambitions (Nilholm 2005). It also reflects the individual versus the social models of disability, which were developed in the mid-1970s by the Union of the Physically Impaired Against Segregation and popularized by the British sociologist Mike Oliver in the early 1980s (Oliver, 2013). The point of pursuing a social rather than medical model of disability was to stimulate professionals to work more from the standpoint that people are not disabled by handicap but by disabling barriers they face in society.

Even a cursory glance at how education is presently organized and what sort of expertise tends to be applied to problems of fit between children and schooling makes it clear that the biomedical model dominates in present inclusive education, even though it is based on the conflicting reasoning of first excluding a child by labelling it as disordered (e.g., ADHD, autism, ODD) and then take this disorder as a reason for inclusion (Dalkilic and Vadeboncoeur, 2016). The language used to describe problems in the classroom influences teachers’ expectations and interactions with pupils (Heagele and Hodge, 2016). When child behavior problems are perceived to be the result of a neurobiological disorder that causes “symptoms” like hyperactivity, teachers may feel less responsible and self-efficient than when behavior is viewed as the result of many factors, including interactions that take place in the school context (Meerman et al., 2017).

Following a 1978 report by Mary Warnock in the UK (Warnock, 1978) that introduced the term special educational needs (SEN), the educational sciences have strived to replace a discourse of social misfits, deviancy and disorder with a discourse that emphasizes that children’s needs are either ordinary and so fully met by regular educational provisions, or special and thereby requiring substantive additional effort on the part of teachers. Supposedly, the question should no longer be what the child *has*, but should focus on what the child *needs* (Warnock, 2005). However, making the distinction between ordinary and special needs still involves highly normative and subjective (that is: *pragmatic*) value judgements of what is normal and what is deviant. The judgment also still presupposes that there is suitably independent, reliable, equitable and widely recognized professional expertise on hand to make safe judgments about who is special and who is not. Further, this distinction still rests essentially in (and perhaps even naturalizes) a process of categorization and the labelling of children. Aside from this being potentially stigmatizing, it reinforces and perpetuates the idea of education performing an important sorting function in the social system (Abbs, 1994; Luhmann and Schorr, 2000), with the power to predetermine life courses. In light of the need for independent and dependable judgment across categories that are both stable and able to generate widespread social assent, it is hardly surprising that the “special need” rhetoric now commonplace across education systems the world over has itself too come to be based on the biomedical model with its individualistic, psycho-medical, “natural kind” assumptions about the nature and origins of disability and difference, in which all the problems are explained by the individual’s biological or somatic deficits (Vehmas, 2010). Hence, the field of special education has itself contributed to the psychiatrization and educational displacement of children. The community, relational or social disability approach of inclusive education, in which schools are organized in such a broadly accommodating and welcoming manner that special education needs cease to exist—and as important, mirror the wide variation among children at all levels of school staffing—still seems far away from todays’ reality (Oliver, 2013).

## Teachers With Special Needs

If one wished to pursue a community approach of inclusive education, and leave behind the trap of biomedical reasoning in which some children are first singled out as unacceptably different, then labelled with having special needs and then made a target for educational inclusion, a simple change in language might be a good start. The change proposed here is to replace the common phrase “pupils with special needs” by “teachers with special needs”. A discourse that focuses on the needs that teachers encounter while addressing specific problems in their class, shifts the focus from considering disorders within children towards problems that teachers *de facto* have with educating some pupils. This may counterbalance the rise in confirmed psychiatric diagnoses in children and facilitate implementation of the community approach of inclusive education.

A second advantage of speaking of teachers with special needs has to do with the connotation of the word “special”. While “special” can contain either a positive or a negative value judgement, in its connection with the phrase “children with special needs” it usually refers to an undesirable characteristic or way of functioning of the child (Wilson, 2002). Would we use it for the needs of teachers however, special might point in a more neutral or even positive direction, for example towards making a challenging job successful or having an optimistic attitude towards solving classroom problems. To make true inclusive education happen, teachers and education professionals generally might be encouraged to think and communicate about what they need in order to realize an inclusive educational environment, one in which all or most children can flourish. Hence, with the special needs we propose, teachers have concern for their need for training, coaching and development as a professional in the context of particular challenges to successful teaching.

A last important potential benefit of adopting the proposed change in language would be the agency and responsibility it gives back to teachers. While individualistic medical assumptions and language disempower teachers and ask them to accept recommendations and conclusions suggested by non-educational specialists such as psychiatrists and doctors, speaking in terms of teacher needs when dealing with problems in the context of the classroom brings the agency and responsibility back to them and to pedagogy (Meerman et al., 2017). The dominant biomedical framing of emotional, behavioral and learning problems diverts attention and resources towards biomedical professionals, at the expense of educational professionals. Those resources might instead be available to education professionals, such as teachers, who are directly involved in the education and development of children on a daily basis.

## Policy and Practice

Internationally agreed goals of educational equity are captured in “school for all” and inclusive education policy, as well as in the UN’s 4th sustainable development goal, which is about access to quality education for all. With respect to educational policy, we note that our call to arrest the growing trend towards psychiatrization and medicalization of child behavior, implies that no major change in policy direction is necessary. Instead of altering policy, we suggest rigorously moving the focus of educational policy implementation away from conceiving of children as having educational needs, to recognizing that children, whatever their individual characteristics and capacities, all have exactly the same access and participation rights. It is education systems and schools that need to deliver parity, and towards that end teachers need to develop special capacities. Our call is therefore not to change policy, but to attend far more critically to the true object of that policy.

This highlights the contrary effects of what the biomedical language that is presently used foregrounds—namely, individual children being labelled as needy—and what that language hides: that education systems, schools and teachers need to develop a special capacity for educational inclusion. Within a community approach of inclusive education the child is not fit to the demonstrable needs of the education system, but professional capacities in schools are raised towards teaching that better fits ever more diverse classrooms.

Our proposal to re-center the meaning of special education on the problems that schools apparently have with educating some children may seem radical or even reactionary to some supporters of special or inclusive education. Yet this idea is neither without precedent nor without good practice. Existing descriptions of educational consulting and schools-based intervention have centered on the assumption that classroom problems are substantively inherent in how schools and classrooms work, in the roles that teachers play and in the assumptions they make (Dinkmeyer et al., 2016). Similarly, in the Netherlands a national network of school support services have long included a form of educational consulting whereby questions that teachers have about teaching pupils are consistently treated as issues of pedagogy and didactics. It is thought that this consultatory foregrounding of pedagogy and didactics are part of a prevention strategy or “good care perspective” aimed at resolving classroom problems before they become a hindrance (Meijer, 2019). A key practice component entailed in the Dutch “CLB” approach (*consultatieve leerlingbegeleiding*, or consultative learner guidance) is that trained coaches guide teachers into framing all discussion of problems that teachers report to them as challenges to professional teaching in a given classroom context. Hence, we could also name this practice Consultative *Teacher* Guidance. The coaching focuses on alternative choices that might be made in pedagogy, didactics, resources, classroom management, and so on—instead of supposing that problems originate in the traits of a particular pupil. The guiding assumption is that a classroom of pupils will inevitably entail a mix of mental and physical traits, while an exciting challenge of teaching is precisely to meet pupils’ learning with optimally adjusted classroom practice even so. The value of this kind of approach was confirmed in empirical studies that assessed the role that educational consultation can play in resolving problems that arise between pupils, parents and educators (Sheridan et al., 1990; Elliott and Sheridan, 1992), including such consultation providing a solid basis for standards of accountability that schools have for educating all pupils (Roach and Elliott, 2009).

## Discussion

We have argued that the rise in childhood disorders will in part be attributable to the widespread support there is in contemporary education praxis for biomedical views on children who are taken to deviate substantively from implicit norms set for standard (as opposed to special) educational effort. This routine sorting of children in the education system into supposedly “normal” children and “special” children (or children with special needs) has historical antecedents and has long made special education a growth sector (Dekker, 2009; Tomlinson, 2012). Yet with the rise of clinical psychiatry and diagnosis, this sorting mechanism has been given a new biomedical foundation and warrant, and given it entirely new growth potential. The symbiosis that has developed, between the psychiatric diagnosis of supposed childhood mental disorders and the sorting of children across regular and special education, has most likely contributed to inflating the numbers of children classified with mental disorders, as well as increased the amount of childhood psychotropic drug prescriptions in the last few decades.

Locating the problems that teachers have with educating some children in an expanding range of individual conditions may be considered progressive in clinical or medical terms, but the consequence of doing so is that children become the sole owners of what are in essence pedagogical issues and challenges in teaching. Diagnosing children with mental disorders inevitably involves a stigmatizing form of subjecting children to potentially lifelong treatment or management of internalized mental conditions. There is meantime little evidence that raising teacher’s awareness of supposedly neuropsychiatric conditions in fact brings about more successful educational inclusion, and indeed regular school teachers generally remain weary of including children with more severe conditions in their classrooms (Pijl, 2010; Saloviita, 2020).

One way out of the far-reaching consequences of present high levels of psychiatric diagnosis of mental disorder in children is to explicitly recognize that education systems sort children by the level of special effort that teachers need to make in educating them. A first step in reversing the psychiatrization of children in education is therefore to recognize that teachers have special needs. Depending on the level of variation among pupils in a given classroom, they may need particular support in order to do inclusive education well. This acknowledges that teachers are likely to face special teaching problems that need then to be addressed with additional resources, effort or professional development. In a *truly* inclusive education system, no child has special educational needs. Instead, teachers are enabled to muster the special powers that they may sometimes need in order to support and nurture every child’s capacities for learning equally, while valuing childhood diversity. Our proposal of shifting the attention in policy and discourse from pupils to teachers having special needs draws a principled pedagogical conclusion from an undesirable state of affairs: the increasing reification of biomedical knowledge is making individual pupils, rather than school communities, the stigmatized owners of problems that arise in education and the education system.

## Data Availability

The original contributions presented in the study are included in the article/supplementary material, further inquiries can be directed to the corresponding author.
